# Loot box purchasing and indebtedness: The role of psychosocial factors and problem gambling

**DOI:** 10.1016/j.abrep.2023.100516

**Published:** 2023-10-13

**Authors:** Anu Sirola, Jussi Nyrhinen, Julia Nuckols, Terhi-Anna Wilska

**Affiliations:** The Department of Social Sciences and Philosophy, University of Jyväskylä, Finland

**Keywords:** Gambling, Gaming, Indebtedness, Loneliness, Loot box, Resilience

## Abstract

**Introduction:**

Loot boxes are increasingly common random-reward monetization mechanisms in digital games. They are popular among gamblers and pose various risks due to their gambling-like nature, but little is known about psychosocial vulnerabilities and financial consequences of purchasing them. This article examined psychosocial associations with self-reported increase in loot box purchasing and indebtedness among past-year gamblers during the COVID-19 pandemic.

**Methods:**

Cross-sectional survey data were collected in April 2021 from Finnish, Swedish, and British past-year gamblers aged 18 to 75 (n = 2,022). Measures of loneliness, psychological resilience, and problem gambling were studied in relation to loot box purchasing and indebtedness. Structural equation modeling was used as an analytical technique.

**Results:**

Loneliness was positively associated with self-reported increase in loot box purchasing. No evidence was found regarding the protective role of psychological resilience in loot box purchasing. Increased loot box purchasing was associated with problem gambling. Problem gambling mediated the relationship between loot box purchasing and indebtedness.

**Conclusions:**

The findings bring valuable insight into the psychosocial vulnerabilities and financial consequences in loot box purchasing. Loot box purchasing can add to one’s financial strain particularly among vulnerable individuals such as problem gamblers, making it crucial to regulate such monetization practices.

## Highlights

• Loot box behavior was surveyed among past-year adult gamblers (n = 2,022)
• Loneliness was associated with increased loot box purchasing.
• Psychological resilience did not protect from increased loot box purchasing.
• Increased loot box purchasing was associated with problem gambling.
• Problem gambling mediated the association between loot box purchasing and indebtedness.

## 1 Introduction

Monetization mechanisms are highly prevalent revenue models in digital games, providing means to enhance one’s gaming experience by using real money. Concerns have been raised particularly in relation to ‘loot boxes’ that present a controversial form of in-game purchases in pursuit of randomized rewards such as weapons or cosmetic features (Brooks and Clark, 2023, Drummond and Sauer, 2018). Most of the top-grossing smartphone games include this feature and more than 70% of desktop video games provided on the Steam platform obtain revenue via loot box purchasing (Wardle and Zendle, 2021, Zendle et al., 2020). The chance-based nature of loot boxes is often juxtaposed with mechanisms of gambling, and these gambling-like mechanisms make them potentially addictive for players (Brooks and Clark, 2019, Delfabbro and King, 2020, Király et al., 2023, Spicer et al., 2022).

There are complex motivational factors in loot box purchasing (Nicklin et al., 2021), but more research is needed on underlying factors in their spending and consequent harms (Yokomitsu et al., 2021). There are only a few studies on psychosocial factors (Drummond et al., 2022, Etchells et al., 2022, Irie et al., 2022) and economic harms (Carey et al., 2022) associated with loot box spending. Most studies have focused on adolescent players (e.g., Hing et al., 2022, Kristiansen and Severin, 2020, Wardle and Zendle, 2021), but digital gaming is increasingly popular among adults as well (Kinnunen et al., 2020). Some players such as those with psychosocial burdens might be more vulnerable to the risks of loot boxes, but research is needed to understand how these vulnerabilities and risks can manifest during crisis situations such as global pandemic.

The main aim of this paper is to investigate self-reported increase in loot box purchasing during the COVID-19 pandemic from a psychosocial perspective. We specifically look at the role of loneliness and psychological resilience in such behavior. Additionally, we investigate the financial consequences of loot box purchasing and the role of problem gambling in these associations. We utilize data gathered from 18 to 75-year-olds from Finland, Sweden, and the UK. These countries represent culturally relatively similar European countries where gaming and gambling are highly prevalent activities (Kinnunen et al., 2020, Zandt, 2022). Since loot box purchasing is commonly associated with gambling and its disordered forms (Li et al., 2019, Spicer et al., 2022, Wardle and Zendle, 2021, Yokomitsu et al., 2021), we scrutinize this behavior among past-year gamblers who represent a vulnerable subgroup for loot box purchasing and associated harms (Brooks & Clark, 2019). We approach loot box purchasing as a form of problematic behavior because of its gambling-like mechanism (Delfabbro and King, 2020, Spicer et al., 2022) and potential to harm one’s finances (Carey et al., 2022). The pandemic provides a context of a crisis situation that has amplified psychosocial problems and distress among vulnerable individuals (Pallavicini et al., 2022, Savolainen et al., 2022).

Our study aims to create a comprehensive model on both psychosocial vulnerabilities and financial consequences in loot box purchasing. Thereby, our study makes an important contribution to a lack of research on psychosocial factors and brings valuable insight on risks associated with these game mechanisms.

## 2 Theoretical background and hypotheses

### 2.1 Psychosocial perspectives on loot box purchasing

Meaningful social connections are vital for wellbeing (Baumeister and Leary, 1995, Deci and Ryan, 2000). Loneliness is an adverse state of social disconnection and perceived deficiency in one’s relationships (Heinrich and Gullone, 2006, Perlman and Peplau, 1981, Weiss, 1973), associated with a myriad of harms and even premature mortality (Heinrich and Gullone, 2006, Holt-Lunstad et al., 2010). Some individuals try to compensate for loneliness by high engagement in online activities such as online communities or video games (André et al., 2020, Latikka et al., 2022). Many digital games provide means for social interaction and communities (Sirola et al., 2021), and social motives are among the key gaming motives (Wang & Cheng, 2022). In the early onset of the pandemic, the World Health Organization recommended digital gaming as a safe activity to spend time and connect with friends and family (King et al., 2020).

Even though digital games partly mitigated feelings of loneliness and mental distress caused by the pandemic (Mohamed et al., 2022, Pallavicini et al., 2022), online relationships may not fully compensate for loneliness and related distress (Latikka et al., 2022). Studies have found that loneliness is a risk factor for problem gambling (Khazaal et al., 2017, Sirola et al., 2023) and high engagement in digital games (André et al., 2020), but its association with loot box purchasing has not been studied before. Therefore, we hypothesize:

H1 Loneliness is positively associated with increased loot box purchasing.

Several studies have found associations between loot box purchasing and poorer mental health and distress (Drummond et al., 2022, Irie et al., 2022, Li et al., 2019), with the pandemic amplifying such distress and consequent problem behaviors (Pallavicini et al., 2022, Savolainen et al., 2022). Some individuals, however, are more resilient to developing problem behaviors. Psychological resilience refers to individuals’ positive adaptation when facing adverse or unexpected situations, and their ability to bounce back after crises (Bonanno, 2004, Connor and Davidson, 2003, Fletcher and Sarkar, 2013). Psychological resilience is generally perceived as a protective factor, and such evidence is found regarding problematic online gaming (Canale et al., 2019, Yen et al., 2019). Regarding problem gambling, studies have not found evidence on the protective role of resilience among adult gamblers (Mishra et al., 2019, Oei and Goh, 2015, Scholes-Balog et al., 2015, Sirola et al., 2023). To the best of our knowledge, the role of psychological resilience has not been studied in relation to loot box purchasing. Therefore, the following hypothesis is worth testing:

H2 Psychological resilience is negatively associated with increased loot box purchasing.

### 2.2 Loot boxes, problem gambling, and financial harm

Loot boxes are commonly juxtaposed with forms of gambling and generally perceived as a gambling-like activity (Brooks and Clark, 2019, Delfabbro and King, 2020, Spicer et al., 2022). There is robust evidence that loot box purchasing and (problem) gambling are associated (Close et al., 2021, Li et al., 2019, Spicer et al., 2022, Wardle and Zendle, 2021, Yokomitsu et al., 2021; Zendle & Cairns, 2018). It has been suggested that loot boxes attract gamblers due to similar experiences of excitement and anticipation than in gambling (Brooks and Clark, 2019, Delfabbro and King, 2020, Li et al., 2019). Loot boxes also provoke similar physiological reactions than gambling (Brady & Prentice, 2021). For some players, loot box purchasing might act as a catalyst for gambling (i.e., ‘gateway hypothesis’, see (Brooks and Clark, 2023, Delfabbro and King, 2020, Spicer et al., 2022). We hypothesize:

H3 Increased loot box purchasing is positively associated with problem gambling.

Financial motives are typical in gambling and its disordered forms, and gambling can be perceived as a way to earn money to ease financial strain (Hagfors et al., 2022, Tabri et al., 2022). Problem gambling is more common among those of lower income (Hahmann et al., 2021), but gambling can further worsen the situation leading to severe financial problems such as indebtedness (Achtziger, 2022, Håkansson and Widinghoff, 2020, Oksanen et al., 2018). Indeed, financial harm is dominant among problem gamblers (Langham et al., 2015). Thus, we hypothesize:

H4 Problem gambling is positively associated with indebtedness.

As a gambling-like monetary activity, loot box purchasing has the potential to become problematic and contribute to one’s financial harm (Carey et al., 2022). Loot box prices typically vary from a few to tens of dollars, and high-spenders use over $100 per month on loot boxes (Close et al., 2021). Accumulating costs can increase financial strain such as debt problems among financially vulnerable players. In digital games, the monetization practices are made highly attractive for players, representing an addictive element (Kíraly et al., 2023). As King and Delfabbro (2018) argue, these ‘predatory monetization schemes’ are designed to make players both financially and psychologically committed to a game with a purpose of spending more and more money. Similar to problem gambling, players might end up using more money than one could afford. Therefore, we test the following hypothesis:

H5 Increased loot box purchasing is positively associated with indebtedness.

Given that gambling activities and loot box purchasing often co-occur (Li et al., 2019, Spicer et al., 2022, Yokomitsu et al., 2021; Zendle & Cairns, 2018), it is meaningful to scrutinize the role of problem gambling in loot box purchasing and indebtedness. Since loot boxes are particularly attractive among gamblers, it is likely that those who have increased their loot box purchasing during the pandemic have problematic gambling tendencies as well. Loot box expenditure can add to financial strain caused by excessive gambling (Hing et al., 2022), but it might be problem gambling that plays a major role in debt problems among loot box buyers. Therefore, it is meaningful to examine whether and to what extent problem gambling mediates the association between loot box purchasing and indebtedness.

### 2.3 Summary of theoretical framework

**Fig. 1:**
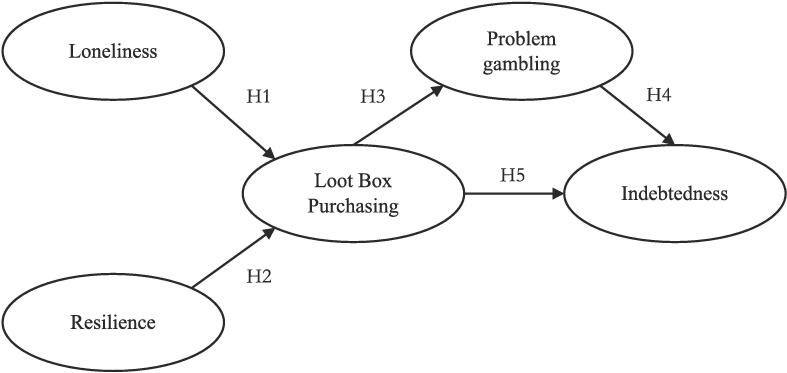
Theoretical Framework and Hypotheses.

Based on our theoretical framework regarding psychosocial vulnerabilities and financial harms in gambling-like behaviors, we propose our research model (see Fig. 1) where loneliness (H1) and (low) psychological resilience (H2) are used as predictors for increased loot box purchasing, and indebtedness is the potential negative financial outcome of loot box behavior (H5). Furthermore, given the research evidence regarding co-occurrence of problem gambling and loot box behavior, we hypothesized an association between loot box purchasing and problem gambling (H3). We also examine the direct linkage between problem gambling and indebtedness (H4).

## 3 Data and methods

### 3.1 Participants and procedures

A total of 2,022 respondents aged 18 to 75 (female 45.1%; mean age = 43.00; SD = 15.24) participated in an online survey from Finland (n = 709), Sweden (n = 714), and the UK (n = 599) in April 2021. The criterion for participating was past-year gambling activity. The survey design and measures were similar in Finnish, Swedish, and English. Data were gathered via a data provider company that used web panel data of volunteer respondents, using random sampling from each country. Data mirrored population estimates regarding gender, age, and living area. Online surveys have been found to be beneficial when collecting data regarding problematic gambling and gaming activities due to the anonymity of responding and respondents’ familiarity with online technologies (Griffiths, 2010).

Data collection was carried out in accordance with the ethical guidelines (Declaration of Helsinki). Respondents were informed about the survey aims, and participation was fully voluntary. Data did not include underaged participants.

### 3.2 Measures

**Loot box purchasing** was measured with a single-item “How have your online consumer habits changed during the coronavirus pandemic regarding the following services in comparison to your previous habits: Loot box purchases in digital games”, with a 5-point response scale *(1 = I have not purchased at all 2 = I have purchased less 3 = the same amount 4 = more to some degree 5 = considerably more*). Use of a single item variable was justified because the statement measured respondent’s own estimation of a past behavior and the risk that one would misinterpret the statement was minimal (see Allen et al., 2022).

**Indebtedness** was measured with a single item “Which of the following statements describe your indebtedness best?” adopted from Wang and Xiao (2009) with the following options: *1 = The payment of bills, instalments and/or loan deductions do not cause me any difficulties and I am able to save money simultaneously; 2 = The payment of bills, instalments and/or loan deductions do not cause me any difficulties, however I am unable to save money simultaneously; 3 = The payment of bills, instalments and/or loan deductions is constantly difficult; 4 = I have received payment requests and have had to pay for late fees, for I have not been able to pay bills, instalments and/or loan deductions when they have been due; 5 = I have a compromised credit rating and/or have been subject to debt recovery procedures.* Thus, higher scores indicate more severe indebtedness. The measure has been utilized in prior studies regarding risky purchasing behavior and financial problems (Nyrhinen et al., 2023).

**Problem gambling** was measured with the Problem Gambling Severity Index (PGSI) that is a psychometrically valid and standardized measure to examine problem gambling in non-clinical context (Ferris and Wynne, 2001, Holtgraves, 2009). The original scale consists of nine items, and it has been widely utilized in survey research in Finland (e.g., Raisamo et al., 2015, Savolainen et al., 2022), Sweden (e.g., Abbott et al., 2018), and the UK (e.g., Orford et al., 2010). Timeframe (past 12 months) was given to reflect pandemic-time problem gambling. The response scale was from 0 to 3 *(0 = never, 1 = sometimes, 2 = most of the time, 3 = almost always),* with higher scores indicating higher problem gambling severity.

**Loneliness** was asked with Three-item loneliness scale that is a shorter version of the full UCLA measure and developed for large-scale survey research (Hughes et al., 2004). The measure has proven to be psychometrically reliable and valid instrument (Hughes et al., 2004, Russell, 1996). A three-part question was asked: Thinking about the past year, how often have you felt: 1) that you lack companionship, 2) left out, 3) isolated from others. Response scale was from 1 to 3 *(1 = hardly ever, 2 = some of the time, 3 = often)*, higher scores indicating higher levels of loneliness. A timeframe (past 12 months) was given to reflect pandemic-time loneliness.

**Psychological resilience** was measured with the 10-item version of the Connor-Davidson scale (CD-RISC) that assesses individual psychological resources to cope with unexpected and stressful situations (Campbell-Sills and Stein, 2007, Connor and Davidson, 2003). The scale has proven to be psychometrically reliable and valid instrument (Windle et al., 2011), also in Finnish (Tourunen et al., 2021) and Swedish contexts (Velickovic et al., 2020). The response scale was from 0 to 4 (*0 = not true at all; 4 = true nearly all of the time*), higher scores indicating higher psychological resilience.

### 3.3 Statistical techniques

To test the conceptual model and proposed hypotheses, Structural Equation Modeling (SEM) with the maximum likelihood estimation method with bootstrapping was employed using IBM SPSS AMOS 26 software. Mediating effect was tested using PROCESS v3.5 by Andrew F. Hayes (Hayes, 2012).

## 4 Results

### 4.1 Measurement model

The measurement scales consisted of 12 items that involved three constructs (Table 1). The measurement model was designed to measure the following latent constructs: *Problem gambling*, *Resilience*, and *Loneliness*. The validity of the measurement model and the unidimensionality of the constructed scales was tested with a confirmatory factor analysis (CFA). Component loadings included in the model were above the threshold value of 0.7 and varied between 0.702 and 0.861. The measurement model fit was found to show an acceptable fit (χ^2^(84) = 297.094, CMIN/DF = 3.537, IFI = 0.986, CFI = 0.986, TLI = 0.982, RMSEA = 0.035, 90% CI [0.031, 0.040], and SRMR = 0.038, RFI = 0.975).

**Table 1:** Constructs and Items.

**Construct**	**Item**	**FL**	***M***	***SD***
Problem gambling *(0 = never; 1 = sometimes; 2 = most of the time; 3 = almost always)*	Have you needed to gamble with larger amounts of money to get the same feeling of excitement?	0.820	0.66	0.90
	Have you borrowed money or sold anything to get money to gamble?	0.861	0.58	0.92
	Have you felt that you might have a problem with gambling?	0.852	0.66	0.94
	Have people criticized your betting or told you that you had a gambling problem, regardless of whether or not you thought it was true?	0.850	0.56	0.87
	Has your gambling caused any financial problems for you or your household?	0.859	0.59	0.92
Loneliness *(1 = hardly ever; 2 = some of the time; 3 = often)*	Lack companionship?	0.727	1.86	0.72
	Left out?	0.840	1.77	0.72
	Isolated from others?	0.705	1.86	0.73
Resilience	I can deal with whatever comes my way.	0.696	2.55	0.92
*(0 = not true at all; 4 = true nearly all of the time)*	I believe I can achieve my goals, even if there are obstacles.	0.741	2.50	0.97
	Under pressure, I stay focused and think clearly.	0.722	2.41	1.01
	I think of myself as a strong person when dealing with life’s challenges and difficulties.	0.702	2.48	1.03
Loot box purchasing *(single-item, scale 1*–*5)*	Loot box purchases in digital games during the COVID-pandemic	n/a	1.91	1.21
Indebtedness *(single-item, scale 1*–*5)*	Which of the following statements describe your indebtedness best?	n/a	2.14	1.18

*Notes*: FL = factor loading, *M* = Mean, *SD* = standard deviation.

The items were also found to converge on their assigned factors (Table 2). The average variance extracted (AVE) exceeded the cut-off value 0.50, and all of the variables' composite reliabilities were between 0.803 and 0.928, indicating strong internal reliability (Bagozzi & Yi, 2012). The measuring of model's discriminant validity was examined using Bagozzi's (1991) and Fornell and Lacker's (1981) AVE methods. Because the square roots of the AVEs for each construct were higher than any construct correlation and the correlations between the constructs were less than 0.60, the square roots of the AVEs demonstrated adequate discriminant validity (Table 2).

**Table 2:** Validity, reliabilities, and intercorrelations.

	**α**	**CR**	**AVE**	**Loneliness**	**PGSI**	**Resilience**
**Loneliness**	0.801	0.803	0.577	0.760		
**PGSI**	0.926	0.928	0.720	0.360***	0.848	
**Resilience**	0.807	0.807	0.512	−0.363***	−0.116***	0.715

*Notes:* *** = p < 0.001; α = Cronbach's alpha; CR = composite reliability; AVE = average variance extracted; PGSI = problem gambling; construct correlations, square root of AVEs (on the diagonal).

### 4.2 Structural model

The structural model fit was assessed through several indices, which indicate a good fit despite the high chi-square value (Schermelleh-Engel et al., 2003). Following the suggested cut-off points by Hu and Bentler (1999), all values indicated a good fit for the model. The values of IFI, TLI, RFI, and CFI were clearly above the cut-off value 0.95 ranging from 0.979 to 0.989; the value of RMSEA was 0.033 (<0.06) and SRMR was 0.054 (<0.08); and the value of CMIN/DF was clearly below the cut-off value of 5 (Hu & Bentler, 1999).

**Fig. 2:**
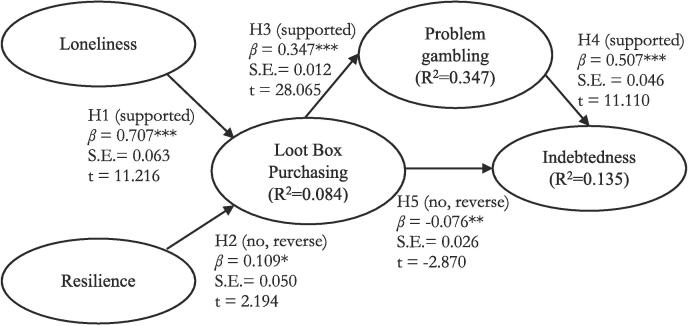
Results of Hypothesis Testing,*Notes:* ***p < 0.001; **p < 0.01; *p < 0.05; DV = dependent variable; IV = independent variable; model fit: χ2 (72) = 223.918; CMIN/DF = 3.154; IFI = 0.989; TLI = 0.985; RFI = 0.979; CFI = 0.989; RMSEA = 0.033; SRMR = 0.054.

The results of hypothesis testing are shown in Fig. 2. With respect to H1, *Loneliness* had a positive association with *Loot Box Purchasing* (*β* = 0.707, t = 11.216, *p* < 0.001). *Psychological resilience* had a positive association with *Loot Box Purchasing* (*β* = 0.109, t = 2.194, *p* < 0.05), which was reversed to H2. With respect to H3, *Loot Box Purchasing* was positively associated with *Problem gambling* (*β* = 0.347, t = 28.065, *p* < 0.001). *Problem gambling* had a positive association with *Indebtedness* (*β* = 0.507, t = 11.110, *p* < 0.001), supporting H4.

Contrary to hypothesis (H5), there was a weak negative association between *Loot box purchasing* and *Indebtedness* (*β* = −0.076, t = −2.870, *p* < 0.01). However, there was a positive indirect link between *Loot Box Purchasing* and *Indebtedness* through *PGSI* (*β* = 0.185, [CI lower 0.157, CI upper 0.214]). Therefore, the positive association between *Loot Box Purchasing* and *Indebtedness* was indirect and mediated through *Problem gambling.*

The conceptual model accounted for 14% of the variance in *Indebtedness*, 35% of the variance in *Problem gambling*, and 8% of the variance in *Loot Box Purchasing*.

We also tested if the model was invariant between genders, countries, and age groups. The results from the chi-square difference test and ΔCFI indicate a significant decrease in fit due to adding in the equality constraints. As a result, we will conclude that we have no evidence of metric invariance between models for different genders, nationalities, and age groups.

## 5 Discussion

This study was among the first to investigate psychosocial factors, namely loneliness and psychological resilience, associated with loot box activity that is considered a gambling-like and a harmful element of video games. Additionally, the study looked at financial consequences and the role of problem gambling in these associations. The COVID-19 pandemic provided a context of a crisis situation that has increased distress and problem behaviors among vulnerable individuals (Pallavicini et al., 2022, Savolainen et al., 2022).

Regarding psychosocial factors, loneliness was associated with increased loot box purchasing, supporting the literature on its risky role in both gambling and gaming behaviors (André et al., 2020, Khazaal et al., 2017, Sirola et al., 2023). This finding is also in line with other studies suggesting that poor psychosocial wellbeing is a risk factor for loot box purchasing (Drummond et al., 2022, Irie et al., 2022, Li et al., 2019). However, to the best of our knowledge, this was the first study to look at the role of loneliness in said purchasing. Interestingly, a study by Etchells et al. (2022) did not find associations between mental wellbeing and loot box purchasing, which indicates that risks and harms of them are likely to work differently depending on the context and underlying risk factors. Since our findings reflect the time of the COVID-19 pandemic, it might be that excessive social isolation has amplified such effects on loot box behavior (Hall et al., 2021). However, due to the scarcity of studies and partially mixed findings regarding the role of psychosocial factors in loot box purchasing, it is important to study these effects in more detail, also in a post-pandemic world.

Evidence was not found regarding the protective role of psychological resilience in loot box purchasing. Instead, there was a small positive association. Even though studies have found an association between higher resilience and lower levels of problematic gaming (Canale et al., 2019, Yen et al., 2019), studies on adult problem gambling have not found such evidence (Mishra et al., 2019, Oei and Goh, 2015, Scholes-Balog et al., 2015, Sirola et al., 2023). Given that loot box opening activates similar physiological and psychological mechanisms than gambling (Brady & Prentice, 2021), this activity might be more akin to gambling than gaming in terms of protective factors, at least among adult players. Also, psychological resilience is not a stable trait but a context-specific process that is affected by various individual and social resources (Lee et al., 2013). Thus, more detailed studies and measurements are needed.

As expected, there was a positive association between increased loot box purchasing and problem gambling. This is in line with an extensive body of prior evidence, showing that loot box spending and problem gambling are likely to co-occur (Close et al., 2021, Etchells et al., 2022, Li et al., 2019, Spicer et al., 2022, Yokomitsu et al., 2021, Wardle and Zendle, 2021; Zendle & Cairns, 2018).

Contrary to expectations, increased loot box purchasing had a small negative association with indebtedness. However, problem gambling mediated the association between said purchasing and indebtedness. Thus, loot box purchasing might not itself contribute to one’s debt problems but rather via the player’s problem gambling tendencies. This is plausible given that loot box purchasing co-occurs with gambling problems (Li et al., 2019, Spicer et al., 2022, Yokomitsu et al., 2021; Zendle & Cairns, 2018), and financial harm is among the most common downsides of problem gambling (Langham et al., 2015). However, loot box purchasing is likely to add to financial harms caused by gambling, and these behaviors might reinforce each other due to their similar psychological and physiological mechanisms (Brooks & Clark, 2019).

From a theoretical perspective, our findings underline the crucial role of meaningful social connection as a key component in wellbeing and a buffer against problem behaviors (Baumeister and Leary, 1995, Deci and Ryan, 2000, Heinrich and Gullone, 2006). High engagement in gambling and gaming activities might serve as a way to mitigate loneliness and related distress (Mohamed et al., 2022, Pallavicini et al., 2022), but social motives can also make players more susceptible to problematic gaming and monetary in-game investments such as loot box purchases (Sirola et al., 2021).

Regarding vulnerabilities, gamblers and problem gamblers are vulnerable subgroups to loot boxes and their gambling-like mechanisms. Loot boxes are prone to activate intuitive and fast purchase decisions for instant rewards without deliberate consideration and reasoning. This is likely to make players both financially and psychologically committed to a game (King & Delfabbro, 2018). Games that contain loot boxes will also often give them to players for free during gameplay as rewards. From the profit aspect of gaming companies, this is a lucrative way to showcase players with the excitement of opening loot boxes and enhancing their gaming experiences, inviting players to purchase more loot boxes with their own funds later on. Since the findings of this study did not find evidence on the protective role of psychological resilience in loot box purchasing, it is worth asking whether the system is so alluring that it ‘bypasses’ such protective factors on an individual level.

Our findings have practical relevance for academics, educators, clinicians, and policy makers. Also companies in gaming and gambling industry should be better informed about the adverse effects of loot box purchasing in order to develop more ethical business models. From the harm perspective, loot box purchasing does not always bring significant harm for wellbeing (see Etchells et al., 2022), but we argue that some players such as lonely individuals and problem gamblers are more vulnerable to these harms. Accumulation of problems and these vulnerabilities might be amplified during stressful situations. We encourage researchers to study the role of psychosocial factors such as loneliness in more detail, also in a post-pandemic world. In the clinical context, it would be important to recognize the overlap between problem gambling and loot box engagement, as well as to recognize risk factors such as loneliness that might drive such behaviors.

Regarding policy makers, we argue that loot boxes need more regulation and effective warning labels in order to protect vulnerable individuals from unintended purchases and money loss (e.g., Drummond et al., 2022). Government bodies around the world have opted to regulate the availability of loot boxes and related activities by displaying the odds of winning and banning certain loot box features (Wardle & Zendle, 2021). However, loot boxes are still widely available (Zendle et al., 2020) and the current warnings are found to be insufficient (Garrett et al., 2023). Game companies mostly profit from vulnerable and excessive purchasers such as problem gamblers (Close et al., 2021), and wide availability of loot boxes can further normalize gambling activities (Spicer et al., 2022). Even though it is important to gain insight on protective factors and educate players about the odds of winning, the game companies should have the main responsibility of protecting players.

### 5.1 Limitations

This study utilized cross-sectional data and thus, assumptions of causal directions are theoretical. Data relied on self-reported measures that are sensitive to biases such as social desirability or biased estimation of one’s behavior. The amount of money used in loot box purchases or the motives behind such activity were not asked. Participants’ other gaming activities, such as specific games played or disordered gaming were not asked. While most of our measures were validated multi-item scales, we utilized single-item measures for one’s loot box activity and economic situation. Single-items can be sensitive to measurement errors, but their use is deemed appropriate when measuring simple constructs (Allen et al., 2022). The data consisted of adult past-year gamblers and thus, results cannot be generalized to younger or non-gambling populations. Data and findings of this study reflect the first year of the pandemic, but the effect of the pandemic cannot be properly detected with cross-sectional data. Thus, the results should be replicated to see whether and to what extent did the pandemic context affect studied associations. We encourage more detailed and longitudinal studies on psychosocial and financial risk and protective factors in loot box purchasing among different age groups and subpopulations.

### 5.2 Conclusion

Loot boxes and their gambling-like mechanisms pose risks for individuals with psychosocial and financial vulnerabilities. Even though loot box purchasing may not itself be a major contributor to one’s financial problems, such behavior can add to one’s financial strain particularly among problem gamblers. The widespread availability and addictive nature of the loot box system makes it crucial to regulate such monetization practices to protect vulnerable individuals such as young people, lonely individuals, and problem gamblers.

## Funding

This work was supported by the 10.13039/501100002341Academy of Finland (decision #335635), the Strategic Research Council established within the Academy of Finland (decision #352544), and JYU.Well, the interdisciplinary community of wellbeing researchers at the University of Jyväskylä.

## CRediT authorship contribution statement

**Anu Sirola:** Investigation, Conceptualization, Writing – original draft, Writing – review & editing, Data curation. **Jussi Nyrhinen:** Investigation, Conceptualization, Writing – original draft, Writing – review & editing, Data curation, Methodology, Formal analysis. **Julia Nuckols:** Investigation, Conceptualization, Writing – original draft, Writing – review & editing. **Terhi-Anna Wilska:** Investigation, Conceptualization, Writing – original draft, Writing – review & editing, Supervision, Project administration.

## Declaration of competing interest

The authors declare that they have no known competing financial interests or personal relationships that could have appeared to influence the work reported in this paper.

## Data Availability

Data will be made available on request.
