# Supplementary material for: Loot box purchasing and indebtedness: The role of psychosocial factors and problem gambling
Source: Addict Behav Rep. 2023 Oct 13;18:100516. doi: 10.1016/j.abrep.2023.100516 (PMC10616135; doi:10.1016/j.abrep.2023.100516)
Supplement: Supplementary data 1 [file mmc1.pdf]

Innolink & ethics in panel data collection

Innolink's processes

Innolink is a member of the Finnish Marketing Research Agencies (FAMRA) and follows the recommendations and guidelines of the association and ESOMAR in its operations.

The member companies of Finnish Association of Marketing Research Agencies (FAMRA) have a strong commitment to quality and professional market research. FAMRA supports it's members in their pursuit to produce reliable research work and sound results. The reliability, professional skills and quality of work are manifested by the high esteem shown by the clients. All the member companies of FAMRA have committed to complying with an ISO 20252 Quality Certification.

The ethical rules listed by the association are:

General

Market research should always be conducted objectively and in accordance with accepted scientific principles.

Rights of data providers

- The participation in the market research is completely voluntary in all stages of the research. Respondents must not be misled when asking for consent to cooperate.
- The identity of the respondents must be kept strictly confidential. If the data provider has given the researcher permission to forward the information in such a form that the identity of the data provider becomes clear from them:
    - the person giving the information must first tell to whom the information is given and for what purpose it will be used, as well as
    - the researcher must ensure that the data are not used for any non-research purposes and that the recipient of the data is committed to complying with the provisions of these basic rules.
- The researcher must take all possible precautions to ensure that the data provider does not suffer direct harm or harm as a result of participating in the marketing research.
- The researcher must be especially careful when interviewing children and young people. In order to interview children, the consent of a parent or guardian must first be obtained.

Professional obligations of researchers

- Researchers must not act in such a way, whether knowingly or negligently, that they bring shame to the field of market research or that the public's trust in market research weakens.
- Researchers must not be allowed to present false information about their own skills or the experience of others working in the organization.
- Researchers should always strive to plan cost-effective and sufficiently high-quality studies and then implement them under conditions agreed with the client.
- Researchers must ensure the safety of all research material in their possession.

Joint rights and obligations of the researcher and the client

- The client does not have the right to receive the names or addresses of the informants unless the researcher has received express consent to this.
- The researcher may not reveal the client's identity (unless required by law) or any confidential information related to the client's transactions to any third party without the client's permission.
- Upon request, the researcher must allow the client to organize fieldwork and data processing quality checks, provided that the client pays all additional costs arising from this.
- The researcher must provide the client with all the necessary technical information about all research projects performed for the client.

Panel provider’s processes

Innolink’s partner in consumer panel data collection is CINT. Cint is a global software leader in digital insights and research technology. It has one of the world’s largest consumer networks for digital survey-based research, with millions of engaged respondents across more than 130 countries.

As a panel operator, CINT does not pass on personal data without the separate consent of the respondent. The operation of the panels is based on opt-in approval, i.e. the respondents have themselves registered as panel members and in this context have given the panel owners their information, which is used to send survey invitations. Respondents can opt-out from the panel at any time. Respondents also make a choice with each research invitation they receive whether to answer the survey or not. CINT does not collect data from children, i.e. below the age limit where parental consent would be required. These age limits may vary slightly from country to country. CINT's privacy notice can be found here: https://www.cint.com/participant-privacy-notice

CINT maintains and ensures quality for every transaction. More about CINT’s commitment to quality: https://www.cint.com/quality

HELSINKI: Itämerenkatu 5, FI-00180 Helsinki, Finland
TAMPERE: Naulakatu 3, FI-33100 Tampere, Finland
TURKU: Köydenpunojankatu 14, FI-20100 Turku, Finland
SEINÄJOKI: Vaasantie 11, FI-60100 Seinäjoki, Finland
VAASA: Hovioikeudenpuistikko 19 A, FI-65100 Vaasa, Finland
Tel. +358 10 633 0200
www.innolink.fi
